# Dose age affect the efficacy of molecular targeted agents in the treatment of hepatocellular carcinoma: a systematic review and meta-analysis

**DOI:** 10.18632/oncotarget.22061

**Published:** 2017-10-19

**Authors:** Jing Du, Ye Mao, Ming Liu, Yan Tie, Hai Huang, Jian Zhao, Zhongzheng Xiang, Di Luo

**Affiliations:** ^1^ State Key Laboratory of Biotherapy and Cancer Center, West China Hospital, Sichuan University, Chengdu, China; ^2^ Collaborative Innovation Center of Biotherapy, West China Hospital, Sichuan University, Chengdu, China; ^3^ Department of Medical Oncology, West China Hospital, Sichuan University, Chengdu, China; ^4^ West China Medical School, West China Hospital, Sichuan University, Chengdu, China

**Keywords:** hepatocellular carcinoma, elderly, randomized controlled trials, meta-analysis

## Abstract

Currently, whether the impact of age on efficacy of molecular targeted agents (MTAs) in the treatment of hepatocellular carcinoma (HCC) patients remains undetermined. We searched databases and abstracts presented at ASCO meeting to identify relevant studies. The endpoints were overall survival (OS) and progression-free survival (PFS). Data were examined using age cutoffs of 65 years. A total of 4,231 HCC patients from eight RCTs were included for analysis, with 1,607 patients aged ≥ 65 years and 2,624 patients aged < 65 years. The pooled results demonstrated that the use of MTAs in patients < 65 years significantly improved PFS (HR 0.69, 95% CI: 0.51–0.95, *p* = 0.023) and OS (HR 0.79, 95% CI: 0.69–0.89, *p* < 0.001) when compared to controls. For HCC patients aged ≥ 65 years, the use of MTAs significantly improved PFS (HR 0.66, 95% CI: 0.53–0.84, *p* = 0.001) but not for OS (HR 0.94, 95% CI: 0.81 –1.09, *p* = 0.41). No publication bias was detected by Begg's and Egger's tests for OS. Therefore, the treatment effect of MTAs on OS might be different in younger and older HCC patients undergoing first-line or second-line treatment, but not for PFS benefit.

## INTRODUCTION

Hepatocellular carcinoma (HCC) ranks the fifth most prevalent cancers worldwide, and is the second most common cause of cancer-related death, with an estimated 748, 300 new liver cancer cases and 695, 900 cancer deaths occurred worldwide in 2008 [1]. The incidence of HCC increases with age and this rate is expected to continue to increase in the upcoming years as the society continues to age [2]. Additionally, HCC usually develops in patients with hepatitis B virus infection, hepatitis C virus infection, or alcoholic liver disease, which develops over a long period of time [3, 4]. And the widely use of anti-viral therapy might further delay the development of HCC. As a result, HCC is generally diagnosed in middle-aged and elderly populations, and management of HCC in elderly patients is becoming a global issue [3, 5].

During the past decade, the emergence of molecularly targeted agents (MTAs) has provided a new promise treatment for HCC. Until now, sorafenib is the only systematic treatment approved by FDA for use in HCC patients [6–8]. Additionally, several novel MTAs have been extensively assessed in clinical trials [9–12]. However, as the stringent enrolment criteria for patients in prospective trials, the enrolled elderly patients in clinical studies are not entirely representative of the overall elderly patient population. In addition, treatment of HCC in older patients may be complicated by several comorbid conditions and greater concomitant medication use compared with younger patients [2, 13]. Other factors such as sensitivity to the toxicity of chemotherapy or molecular targeted agents may also require special consideration. As a consequence, clinical data obtained in a younger population cannot be automatically extrapolated to the great majority of non-selected elderly patients with HCC.

Currently, the concept of “elderly” has become more difficult to define. In general, the chronological age of 65 years- roughly equivalent to retirement age – is currently accepted as a threshold to define an “elderly” person. As the elderly HCC population increases, it is urgently needed to define the best treatment strategy for these patients. In the present, we assess the efficacy of MTAs in the treatment of elderly HCC patients by using age cutoffs of 65 years to determine whether ageing might impact on efficacy of MTAs in this setting.

## MATERIALS AND METHODS

The methods would be described in four steps: definition of the study outcomes, selection of studies, data extraction, and the description of the statistical methods used.

### Definition of outcomes

Treatment with molecular targeted agents (MTAs) was considered as the experimental arms and the other treatments as the standard comparators. The outcomes used were (1) OS, defined as the time from random assignment to death from any cause, censoring patients who had not died at the date last known alive; (2) PFS, defined as the time from random assignment to first documented progression.

### Selection of studies

The Pubmed, Embase, and Cochrane Library electronic databases were search to identify relevant studies of molecular targeted agents as second treatment for advanced HCC (published before December January, 2017). The search was limited to human studies and randomized controlled trials (RCTs). No language restriction was imposed. If more than one publication was found for the same trial, the most recent was considered for analysis.

### Data extraction

Two authors independently extracted the data from included trials. We conducted this meta-analysis based on the Preferred Reporting Items for Systematic review and Meta-Analysis (PRISMA) statement (Supplementary Table 1) [14]. Disagreements between investigators were resolved by discussion and consensus. A standardized Excel file was used for data extraction. The following data were extracted: first author, publication year, the number of enrolled patients and elderly patients, median age, hazard ratios (HRs) with 95% confidence intervals (CIs) for OS and PFS in elderly patients.

### Statistical method

Statistical analysis of the overall hazard ratio (HR) for OS and PFS was calculated using Version 2 of the Comprehensive MetaAnalysis program (Biostat, Englewood, NJ). PFS and OS were considered as time-to-event variables, and therefore were expressed as HRs with 95% CIs for each study. HR > 1 reflected more deaths or progression in MTAs-containing regimens group, and vice versa. Heterogeneity across the studies was assess by using the χ^2^-based Q statistic [15]. The *I*^2^ statistic was also calculated to quantitatively evaluate the degree of inconsistency between trials. We used the Begg and Egger tests to assess the presence of publication bias [16]. Study quality was roughly assessed by using the Jadad five-item scale [17]. All *p*-value of less than 0.05 was considered statistically significantly.

## RESULTS

**Figure 1:**
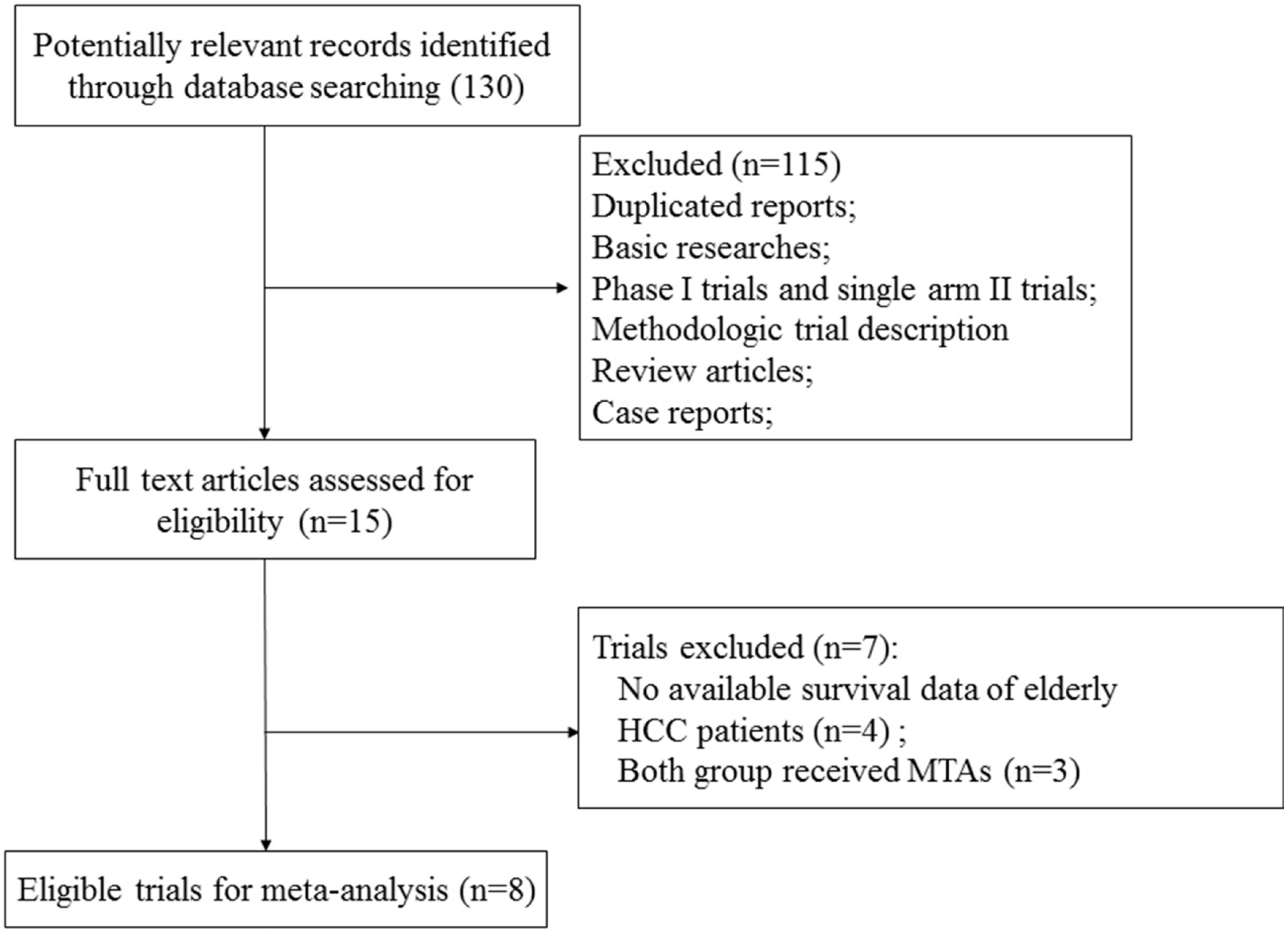
Studies eligible for inclusion in the meta-analysis

Our search yielded 230 clinical studies relevant to MTAs in HCC patients. After reviewing the title or abstract, a total of 8 prospective randomized controlled trials were included for analysis, included 1 phase II trials [18] and 7 phase III trials [19–25]. The flowchart of the search strategy is shown in Figure 1. A total of 4,231 patients were included in the present study. The characteristics of patients and studies were listed in Table 1. The clinical characteristics were generally balanced between the intervention and control arm. The quality of each included study was roughly assessed according to Jadad scale, the median Jadad score of the included studies was 5 (range 3–5).

**Table 1:** Baseline characteristics of eight included randomized controlled trials

Authors/year	Phase	Total	Cutoff of age	No. of patients	Treatment arms	median PFS, m	median OS, m	Jadad Score
Cheng A.L. et al./2009	III	271	≥ 65	32	Sorafenib 400 mg bid po	2.8	6.5	5
			< 65	194	placebo	1.4	4.2	
Kudo M.et al./2011	III	458	≥ 65	152	Sorafenib 400 mg bid po + TACE	5.4	29.7	5
			< 65	306	Placebo + TACE	3.7	NR	
Kudo M.et al./2014	III	502	≥ 65	159	Brivanib 800 mg qd po	12	26.4	5
			< 65	343	Placebo	10.9	26.1	
Zhu Y.X. et al./2014	III	546	≥ 65	298	Everolimus 7.5 mg/d	3	7.6	5
			< 65	248	placebo	2.6	7.3	
Bruix J. et al./2015	III	1114	≥ 65	370	Sorafenib 400 mg bid po	8.5	NR	5
			< 65	744	Placebo	8.4	NR	
Kang Y.K. et al./2015	II	202	≥ 65	85	Axitinib 5 mg bid po	3.6	12.7	3
			< 65	117	Placebo	1.9	9.7	
Zhu A.X. et al/2015 (REACH)	III	565	≥ 65	253	Ramucirumab 8 mg/kg	2.8	9.2	5
			< 65	312	Placebo	2.1	7.6	
Bruix J. et al./2017	III	573	≥ 65	258	Regorafenib 160 mg po	3.1	10.6	5
			< 65	315	Placebo	1.5	7.8	

Abbreviations: OS, overall survival; PFS, progression-free survival; TACE, transcatheter arterial chemoembolization; NR, not reported

### Progression-free survival

**Figure 2:**
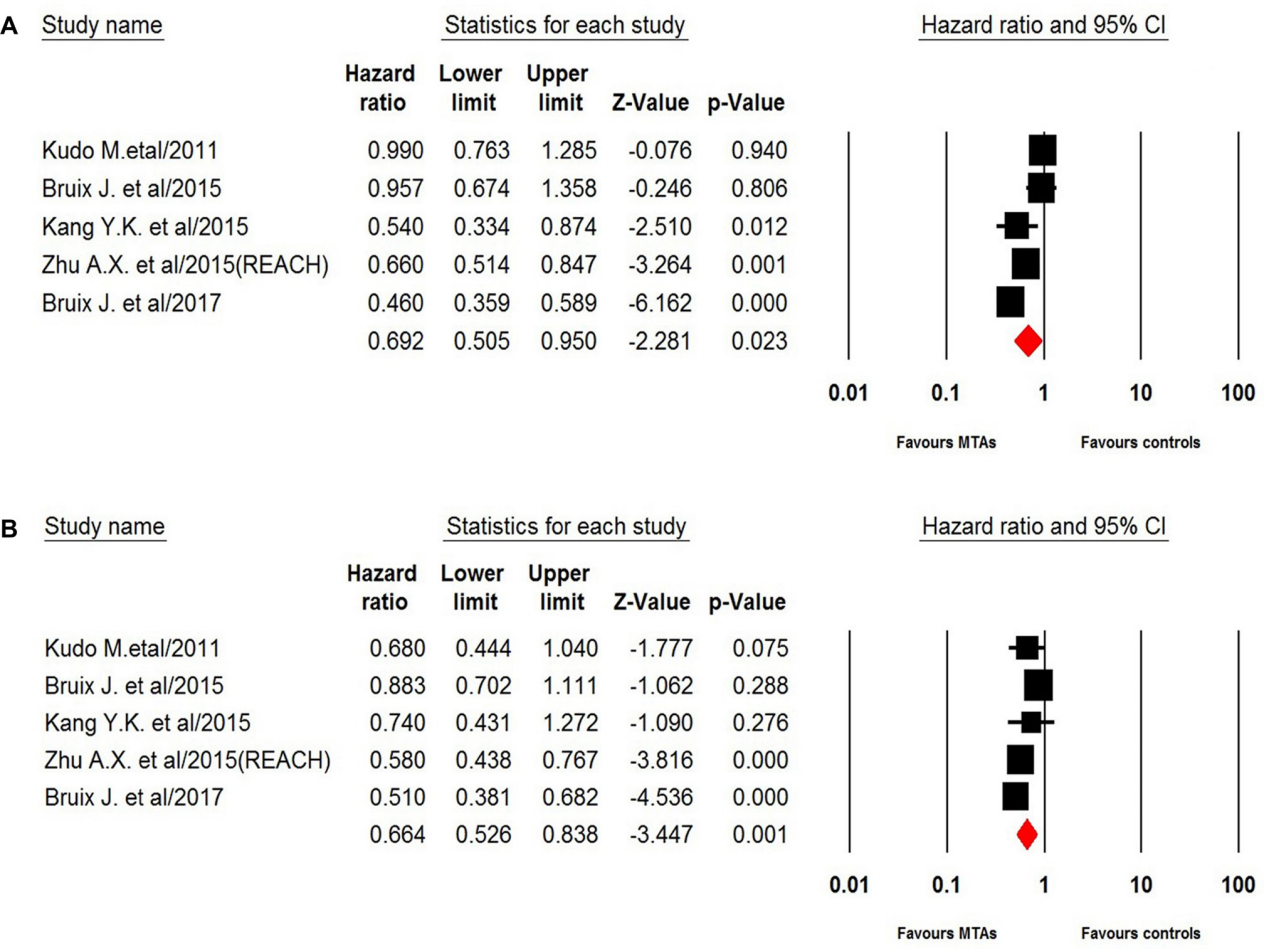
Random-effect model of hazard ratio (95% CI) of PFS associated with MTAs in young or elderly patients

Five trials of the eight trials reported PFS data in the study patients. The pooled results of these studies indicated that the MTAs-containing regimens significantly improved PFS in young HCC patients giving HR 0.69 (95%CI: 0.51–0.95, *p* = 0.023, Figure 2A), compared with non-MTAs containing regimens. There was significant heterogeneity among included trials (*I*^2^ = 82.0, *Q*-value = 22.2, *p* < 0.001), and the pooled results was performed by using random effect model. For patients aged ≥ 65 years, the use of MTAs in HCC also significantly improved PFS in comparison with controls (HR0.66, 95%CI: 0.53–0.84, *p* = 0.001, Figure 2B), and there was moderate heterogeneity among included trials (*I*^2^ = 60.3, *Q*-value = 10.1, *p* = 0.039).

### Overall survival

**Figure 3:**
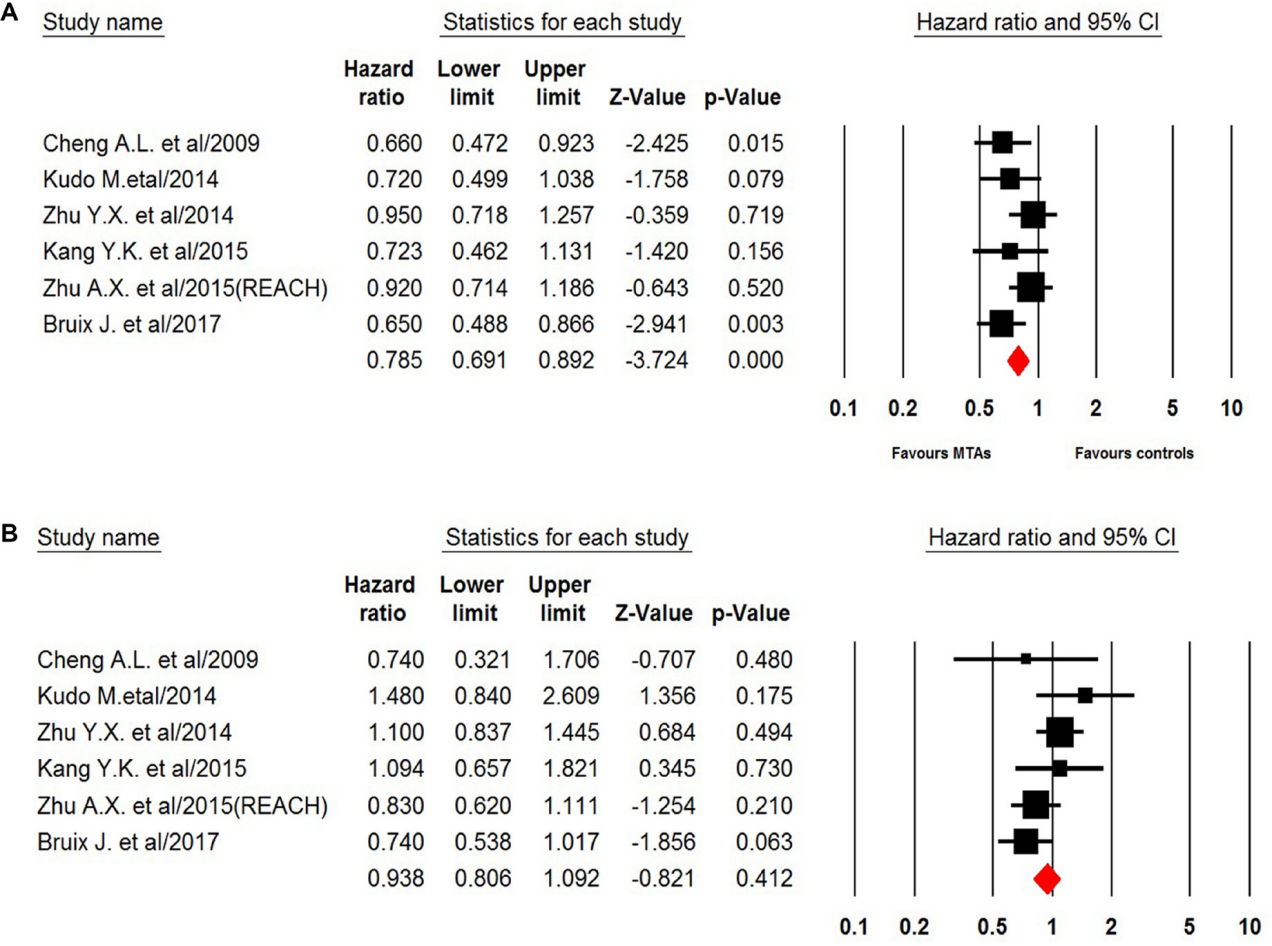
Fixed-effects model of hazard ratio (95%CI) of OS associated with MTAs in young or elderly patients

Six trials of the eight trials reported OS data of elderly patients. For patients aged < 65 years, the pooled results demonstrated that MTAs-containing regimens significantly improved OS in comparison with non-MTAs containing regimens (HR 0.79, 95% CI: 0.69–0.89, *p* < 0.001, Figure 3A) using a fixed-effects model (*I*^2^ = 20.8, *Q*-value = 6.3, *p* = 0.28). However, the use of MTAs did not improve elderly (aged ≥ 65 years) HCC patients’ outcomes (HR 0.94, 95% CI: 0.81–1.09, *p* = 0.41, Figure 3B) by using a fixed-effect model (*I*^2^ = 31.2, *Q*-value = 7.27, *p* = 0.20). Begg's test and Egger's test revealed no evidence of obvious publication bias (*p* = 0.78 and *p* = 0.94, respectively).

## DISCUSSION

In the past years, the mechanisms of hepato-carcinogenesis have been elucidated, and the involvement of a number of pathways, including angiogenesis and dysregulated cell cycle control, have been demonstrated, which leads to the introduction of some novel agents. Indeed, some of these MTAs represent the most promising treatment strategy to improve outcome for patients with advanced HCC. A previous meta-analysis conducted by Niu M. et al. [26] showed that the use of targeted therapies in HCC patients significantly improved survival in comparison with placebo. However, there is limited data specifically focusing on the efficacy of targeted agents in elderly patients with HCC, and most of these published data are retrospective studies with controversial results. For example, Wong et al. [27] reported that the efficacy of sorafenib in elderly patients (≥ 70 year) was comparable to that of young patients (median OS, 5.32 versus 5.16 months), and they also found that there were no differences in grade 3 or 4 toxicities between elderly and non-elderly patients. Similarly, Costanzo et al. [28] also found that there were no significant differences in OS and time-to progression between elderly and non-elderly HCC patients. Conversely, several investigators have advised against the use of sorafenib in elderly HCC patients. Morimoto et al. [29]. found that older age was a significant prognostic factor for poorer survival in a multivariate analysis (HR 0.237, *p* = 0.018). In addition, treatment discontinuation as a result of sorafenib was more frequent in elderly HCC patients (41.7%) when compared to non-elderly patients (15.0%). In consistent with these results, Edeline et al. [30] also found that definitive cessation of sorafenib treatment was observed more frequent in elderly group than in non-elderly group (45.1% versus, 24.4%, *p* = 0.014). As a result, the role of MTAs in the treatment of elderly patient with HCC remains undetermined, we thus perform the present study to investigate the overall efficacy of MTAs in the treatment of this population group.

Our systematic review is, as far as we known, the first systematic review to specially assess the efficacy of MTAs in the treatment of elderly HCC patients. Our study includes a total of 4, 231 HCC patients from eight RCTs were included for analysis, with 1,607 patients aged ≥ 65 years and 2, 624 patients aged < 65 years. Our results demonstrate that the use of MTAs improves PFS in both younger (HR 0.69, 95% CI: 0.51–0.95, *p* = 0.023) and elderly (HR0.66, 95% CI: 0.53–0.84, *p* = 0.001) patient. In addition, the use of MTAs significantly improves overall survival in younger HCC patients (HR 0.79, 95% CI: 0.69–0.89, *p* < 0.001), but not for elderly HCC patients (HR 0.94, 95% CI: 0.81–1.09, *p* = 0.41). However, there is significantly heterogeneity among included studies when analyzing the above endpoints. One possible explanation for this heterogeneity is that our study pooled studies across different lines of therapy investigating MTAs with different modes of action (ramucirumab, regorafenib, axitinib, everolimus, sorafenib, and axitinib). The findings of this study suggest that the treatment effect of MTAs on OS might be different in younger and older HCC patients undergoing first-line or second-line treatment, but not for PFS benefit. Further studies are still needed to assess the role of MTAs in the treatment of elderly HCC patients.

Several limitations exist in this analysis. First of all, this is a meta-analysis at study level. We could not obtain individual patient data from the publication, thus we could not incorporate patients variables into the analysis. For instance, elderly patients are more likely to have comorbid conditions, and we are unable to investigate whether the survival benefit is similar in elderly patients with or without comorbid conditions. Second, none of the included trials report the toxicities of MTAs in elderly patients. Thus, we could not answer whether the use of MTAs in this patient population would increase the toxicities in comparison with controls. Thirdly, there is considerable heterogeneity among the included studies, because different targeted agents are included for analysis. Fourthly, there is still no general agreement on the definition of the elderly population. In the present study, all for the included trials define elderly patients as more than 65 years. Finally, in the meta-analysis of published studies, publication bias is important because trials with positive results are more likely to be published and trials with null results tend not to be published. Our research detects no publication bias using Begg and Egger tests for OS but not for PFS. In the present, we detect no publication bias using Begg and Egger tests for OS.

## CONCLUSIONS

The findings of this study suggest that the treatment effect of MTAs on OS might be different in younger and older HCC patients undergoing first-line or second-line treatment, but not for PFS benefit. Further studies are needed to clearly investigate the role of MTAs in the treatment of elderly HCC patients.

## SUPPLEMENTARY MATERIALS TABLE




