# Supplementary material for: Dose age affect the efficacy of molecular targeted agents in the treatment of hepatocellular carcinoma: a systematic review and meta-analysis
Source: Oncotarget. 2017 Oct 19;8(60):102413–9. doi: 10.18632/oncotarget.22061 (PMC5731966; doi:10.18632/oncotarget.22061)
Supplement: Supplementary file 1 [file oncotarget-08-102413-s001.pdf]

Dose age affect the efficacy of molecular targeted agents in the treatment of hepatocellular carcinoma: a systematic review and meta-analysis

SUPPLEMENTARY MATERIALS

Supplementary Table 1: PRISMA 2009 Checklist. See_Supplementary_Table 1
